# Death by nitrogen anoxia: On the integrated physiology of human execution

**DOI:** 10.1113/EP091836

**Published:** 2024-03-29

**Authors:** David C. Poole, Damian M. Bailey

**Affiliations:** ^1^ Departments of Kinesiology, Anatomy and Physiology Kansas State University Manhattan Kansas USA; ^2^ Department of Anatomy & Physiology Kansas State University Manhattan Kansas USA; ^3^ Neurovascular Research Laboratory, Faculty of Life Sciences and Education University of South Wales Glamorgan UK

## 1 INTRODUCTION

In opposition to any stated opinion that the death penalty and state execution are somehow humane, we demonstrate herein that the latest method, nitrogen anoxia, invoked by the state of Alabama is inherently inhumane. From a respiratory and cerebral bioenergetics approach, we show that both the methods used and their application are flawed with physiological and forensic misconceptions. We are in lock‐step with the leagues of death penalty opponents and consider that its use should be discontinued immediately. However, given that it appears that several US states will simply not stop killing their citizens, based on physiological considerations, might they at least consider a less painful method than nitrogen anoxia, although none may be considered humane?

## 2 THE CHEQUERED HISTORY OF EXECUTION

The Athenian philosopher Socrates, an unconventional thinker who openly challenged the legitimacy and authority of the warlike gods promoted by the state, was found guilty of corrupting the youth with his ideas. Sentenced to death in 399 BC, he was required to carry out his own execution by consuming a deadly concoction thought, by some, to contain the poisonous plant *Conium maculatum*, known popularly as hemlock, causing death by respiratory paralysis and suffocation. Socrates’ last request was for an offering to thank the physician god Asklepios for providing such an effective poison (Bailey, 2018). With rotten descent of democracy into mob rule, Athens lost one of its greatest thinkers owing to a perceived threat. Fast forward almost two and a half millennia to the case of Kenneth Smith, an alleged contract killer in the state of Alabama (Andone et al., 2024) who became the first person known to be executed by nitrogen anoxia, and you will be forgiven for thinking that little has changed. With a misinformed, some may say delusional, focus on improving the means (supposedly more humane) rather than questioning its underlying ethos, Smith's case achieves nothing more than to highlight the ongoing barbarity of state execution, an atavistic relic from the past with a chequered history.

In bygone eras, public executions by extended torture, crucifixion, burning and disembowelling, or by hanging, drawing and quartering, for example, served as painful warnings to the public against bad behaviour or incurring political (as Socrates) or religious disfavour, with death secondary to the infliction of pain and retribution. Society has sought supposedly more humane and dignified means of killing its unwanted citizens, looking to lessen the suffering of the condemned. Practices such as hanging, decapitation (e.g. by sword, Louisette or guillotine), electrocution (proposed by none other than Thomas Edison himself), shooting, gassing or lethal injection of a fast‐acting anaesthetic (sodium thiopental), muscle‐paralysing agent (pancuronium bromide) and cardiotoxin (potassium chloride, reviewed by Quine et al., 1988), either directly or indirectly, arrest O_2_ delivery to the brain, resulting in rapid loss of consciousness and subsequent death.

On Thursday 25 January 2024, Kenneth Smith was executed by being forced to inhale supposedly pure nitrogen gas supplied into a mask (Andone et al., 2024). Smith was pronounced dead at 8:25 pm, and the Alabama Department of Corrections Commissioner, John Hamm, reported that nitrogen was running into Smith's mask for ∼15 min and he thought that Smith held his breath for the initial 4 min. Eyewitnesses observed that Smith seemed to be conscious for ‘several’ minutes into the execution before ∼2 min of shaking and writhing on the gurney followed by several more minutes of deep breathing prior to breathing slowing progressively until it was ‘no longer perceptible for (sic) media witnesses’.

America is unusual among Western countries in still enforcing the death penalty since it was reinstated by the Supreme Court in 1976. Whether the killing of citizens is viewed as just punishment or as a moral, judicial and societal failing, the claim has been made by the state of Alabama that so‐called ‘nitrogen hypoxia’, which is, in fact, nitrogen anoxia, is ‘perhaps the most humane method of execution ever devised’. This statement runs contrary to the opinion of Smith's spiritual adviser, Reverend Jeff Hood, who had watched previous executions by lethal injection, commenting that Smith's death was ‘the most horrible thing I have ever seen’ (Andone et al., 2024).

It is pertinent that the state's published nitrogen anoxia execution protocol is heavily redacted to shield explicit details from public scrutiny. That said, the indication is that room air, containing 20.9% O_2_, balance (78%) nitrogen, is replaced at the turn of a valve by pure (100%) nitrogen. As physiologists, it is our imperative to examine all facets of physiological regulation and how nitrogen anoxia impacts the body until death. Drawing on an extensive literature in both humans and animals (euthanasia), the science of judo's *shime waza* strangles (used also in Brazilian jiu‐jitsu) and fundamentals of systemic and cerebral bioenergetics, this examination provides integrated insights into the events preceding death by nitrogen anoxia. This analysis also brings sharply into question whether, as claimed by the state of Alabama, nitrogen anoxia is the most humane method of execution possible.

## 3 RESPIRATORY PHYSIOLOGY OF NITROGEN ANOXIA

### 3.1 What and where are body oxygen stores, and how long could they last while breathing pure nitrogen?

At rest, the human body uses ∼3.5 mL O_2_/kg/min, known as 1 MET (metabolic equivalent or standard metabolic rate), which equates to ∼0.25 L O_2_/min for a 70 kg individual. As estimated in Table 1, if completely depleted these stores would last 1.55 (total O_2_ stores)/0.25 (1 MET) = 372 s or 6 min 12 s.

**TABLE 1:** Body O_2_ stores.

Compartment	O_2_ concentration (mL/L)	Relevant volume (L)	O_2_ store (L)
Lung (at FRC)	150	2.5	0.4
Blood (arterial)	200	1.5	0.3
Blood (venous)	150	3.5	0.5
Muscle myoglobin	11	30	0.3
Dissolved	0.9	50	0.05
Total O_2_ stores			1.55

*Note*: Values presented are for a 70 kg individual. Calculations assume that blood haemoglobin concentration is 15 g/100 mL, arterial blood is 97% saturated and venous blood is 75% saturated, muscle myoglobin concentration is 0.5 mM, and intramyocyte and ‘average’ tissue/extracellular fluid O_2_ partial pressure is 30 mmHg. Abbreviation: FRC, functional residual capacity.

That said, a substantial portion of these O_2_ stores is not accessible for supporting systemic metabolism. Specifically, breathing pure nitrogen will wash the O_2_ stores out of the lungs into the expiration at a rate dependent upon the extant ventilation and the breathing pattern (i.e., faster washout at higher ventilation and tidal volumes). As the arterial blood becomes progressively more hypoxic, especially below an O_2_ partial pressure ($P_{O_{2}}$) of 60 mmHg (Iturriaga et al., 2021; West, 1995), the peripheral chemoreceptors drive a powerful hyperventilation such that far less than 0.4 L of lung O_2_ stores will be available to support metabolism. This is accompanied by an intense air hunger with involuntary diaphragmatic contractions that signify the physiological break‐point and ensuing struggle phase observed in extreme voluntary breath‐holds and during involuntary suffocation. It is also pertinent that myoglobin stores release their O_2_ only at extremely low values of $P_{O_{2}}$ (myoglobin *P*_50_ is ∼2.5 mmHg), and this O_2_ will not be available to the rest of the organs, including the brain.

Consequently, depending on the ventilatory response, the individual's specific functional residual capacity and whether the room air‐to‐nitrogen switch is made at the end of a quiet exhalation (i.e., at functional residual capacity) and the precise metabolic rate (O_2_ uptake), the accessible O_2_ stores could be expended within 2–6 min or less. However, as seen in the next subsection, while breathing 100% nitrogen the brain will become O_2_ deprived far more rapidly.

The O_2_ expenditure rationale based on Table 1 is broadly consistent with observations in animals that have a higher metabolic rate than humans. Specifically, when Herin et al. (1978) used 100% nitrogen flushing to reduce inspired O_2_ from 21% to <1.5% within 45–60 s, dogs lost consciousness in ∼40 s and were clinically dead, as assessed by flat EEG (80 s), zero blood pressure and lack of spontaneous respiration in 204 s. Initially, the dogs hyperventilated, presumably owing to carotid body stimulation by lowered arterial $P_{O_{2}}$, but subsequently, after the onset of high‐amplitude, slow EEG this ventilatory pattern was not evident. After they became unconscious, some dogs yelped, whereas others gasped, convulsed and/or displayed muscular tremors. These latter behaviours occurred after sensibility had been lost, and they were thus judged to be insensitive to painful stimuli, such as pinching the foot webbing.

### 3.2 Cerebral bioenergetics and vulnerability to failure

**FIGURE 1:**
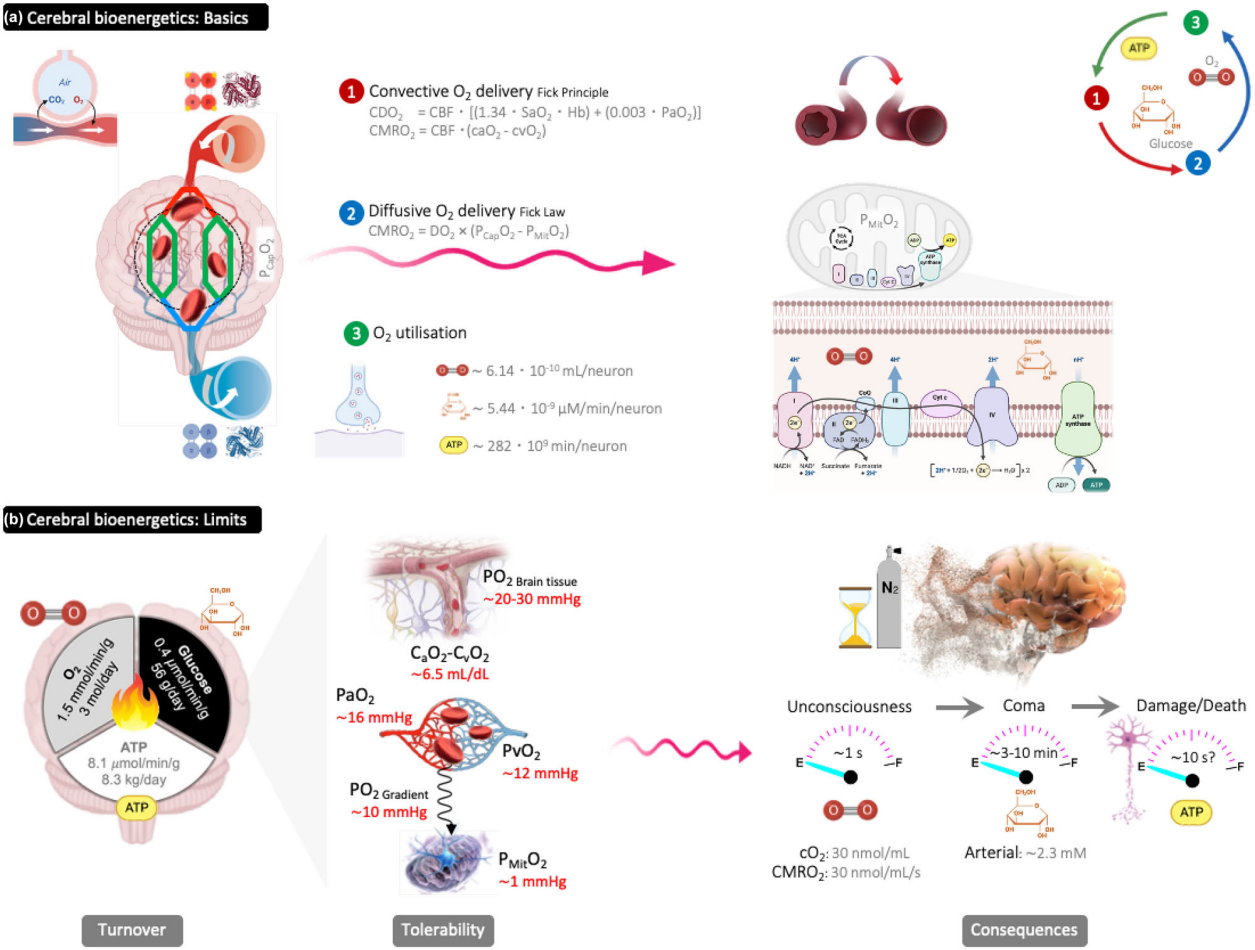
Cerebral bioenergetics: from basics to limits. (a) Schematic diagram highlighting the neurovascular unit and composite interactions between convective (bulk delivery of O_2_) and diffusive (movement of O_2_ from haemoglobin to mitochondria) elements underlying cerebrovascular O_2_ transport and utilization. (b) Given its disproportionately high neuronal ATP turnover to support synaptic transmission combined with limited O_2_/glucose/glycogen reserves, the human brain has evolved exquisite sensitivity to anoxia (pure nitrogen breathing). Note theoretical estimation of critical ‘tolerable’ limits (thresholds highlighted in red) in the cerebral O_2_ cascade thought to precede unconsciousness based on prior boundary calculations (Bailey et al. 2016; Bailey, 2019a). Abbreviations: a, arterial; CBF, cerebral blood flow; CDO_2_, cerebral delivery of oxygen; CMRO_2_, cerebral metabolic rate of O_2_; cO_2_, oxygen content; DO_2_, diffusion incorporating surface area, thickness of the diffusion barrier and O_2_ pressure gradient; Hb, haemoglobin; P_Cap_O_2_, capillary partial pressure of O_2_; P_Mit_O_2_, mitochondrial partial pressure of O_2_; SaO_2_, arterial oxyhaemoglobin saturation; v, venous. Figure created using Biorender, adapted from Bailey (2019a, 2019b) and Bailey et al. (2019).

Unlike most other organs, an evolutionary ‘drive for size’ means that the human brain is committed to a continually active state, relying on a constant supply of blood, given that it has little to no glucose or glycogen reserves and is constrained by a relatively modest capillary density (Bailey, 2016; Bailey et al., 2017). Preservation of cerebral O_2_ consumption is achieved by the maintenance of cerebral O_2_ delivery and involves tight coupling between cerebral blood flow and O_2_ supply/demand, incorporating convective and diffusive components (Figure 1a). Given its meagre energy stores and despite weighing <1/50th of the total body mass, the brain allocates a disproportionate 20%−25% of the basal systemic O_2_ budget (Kety, 1957) to fuel the maintenance of ionic equilibria and uptake of neurotransmitters for synaptic transmission (Figure 1a), with neural tissue ‘costing’ ≤16 times more to maintain compared with skeletal muscle (at rest) and other tissues.

However, its inability to compromise on such an excessive energy budget, with substrate turnover involving a staggering 8.3 kg of ATP/day, equivalent to six times the brain's own mass (Zhu et al., 2018), comes at a cost, rendering the brain exquisitely vulnerable to bioenergetic failure (Figure 1b). Simple division of its metabolic rate(s) by energy content (Figure 1b) highlights how quickly the meagre fuel reserves would be depleted if exposed to an anoxic nitrogen inspiration such that brain O_2_ delivery ceases. The first ‘fuel’ to suffer is O_2_, with its limited reserves depleted in a single second, followed swiftly by its ‘sister substrate’, glucose. Note the ‘critical’ values of $P_{O_{2}}$ and corresponding concentrations that serve as theoretical boundary thresholds, preceding loss of consciousness, coma and, ultimately, neuronal damage and death. The cerebral cortex, hippocampus, basal ganglia and cerebellum are especially sensitive to the ravages of anoxia (Figure 1b).

### 3.3 Physiological responses to breathing pure nitrogen

The first inhalation of pure nitrogen will lower the alveolar $P_{O_{2}}$, impair lung–blood O_2_ diffusion and progressively compound arterial hypoxaemia (i.e., low arterial $P_{O_{2}}$ and O_2_ content). A few seconds downstream of the lung, at the bifurcation of the common carotid arteries, lie the carotid bodies, which are the only chemoreceptors that respond to low arterial blood $P_{O_{2}}$ by stimulating ventilation. When the arterial $P_{O_{2}}$ falls from 90–100 mmHg in normoxia to <60 mmHg while breathing pure nitrogen, the carotid bodies stimulate a marked hyperpnoea and concomitant dyspnoea (Iturriaga et al., 2021; Ward & Whipp, 1989). This will increase ventilation further, effectively helping to wash out any O_2_ remaining in the lungs and reducing arterial $P_{O_{2}}$ to a greater extent. The $P_{O_{2}}$ in the lung might fall well below that in the venous blood, causing a paradoxical blood‐to‐lung movement of O_2_ and accelerating the lowering of arterial $P_{O_{2}}$ (Ernsting, 1963). As demonstrated from animal studies (in cats, rabbits and dogs) of severe hypoxia (not anoxia as with 100% nitrogen breathing) when the O_2_ concentration in the chamber was lowered rapidly, the vast majority of animals collapsed within 60 s, resulting in reflex anoxic myoclonus and seizures and respiratory arrest within 120 s thereafter, followed swiftly by circulatory arrest at 360 s (Quine et al., 1988). The convulsions followed a patterned sequence, with extensions of the front legs and flexion of the hind legs, occasionally accompanied by vocalizations. In humans, Ernsting found that breathing pure nitrogen induced collapse, convulsions and unconsciousness within 17–20 s (Ernsting, 1963) which was accompanied by a 5‐ to 6‐fold elevation in ventilation and increase in heart rate and blood pressure. There will probably also be a substantial sympathetic (flight or fight) response raising blood catecholamines. Thus, together with any overt struggling, the O_2_ uptake demand will increase owing to elevated respiratory and cardiac muscle work and the metabolic stimulation from the rise in blood catecholamines. These demands will shorten the time elapsed to critical depletion of body O_2_ reserves (Figure 1b).

## 4 LEGAL AND FORENSIC MISINFORMATION

Given the politically charged nature of exacting the death penalty and that it is clearly an infraction of the Hippocratic oath, it is not surprising that legal reports and the medical literature are rife with misinformation. For instance, a report entitled ‘*Nitrogen‐induced hypoxia as a form of capital punishment*’ was instigated by Oklahoma State Representative Mike Christian (Copeland et al., 2015). Written by two doctors of jurisprudence (Michael Copeland and Christine C. Pappas) and a Masters degree holder in human resources/criminal justice (Thomas M. Parr), this document claimed that ‘nitrogen hypoxia’ was humane and assured a ‘quick and painless death’.

Copeland et al. (2015) based their report, in part, on the work of Ernsting (1963) and posited further that ‘inhalation of only 1–2 breaths of pure nitrogen will cause a sudden loss of consciousness’. Considering that the one to two breaths were normal tidal volumes of 0.5 L, these would dilute the initial ∼16% O_2_ in the lungs to 14% on the first breath and 12% on the second breath. This might lower arterial $P_{O_{2}}$ from its normal 95–100 mmHg to ∼50 and 36 mmHg, respectively, but would certainly not reduce the arterial O_2_ to the level that might result in a loss of consciousness. However, both values are consistent with providing an intense respiratory stimulation and dyspnoea via the carotid bodies. Moreover, at a normal breathing frequency of ∼15 breaths/min, the 17–20 s elapsed before Ernsting's subjects lost consciousness allows for at least four or five breaths, and far more if the subject is hyperpnoeic and hyperventilating owing to nervousness and the carotid body response to the falling arterial $P_{O_{2}}$.

The report by Copeland et al. (2015) seems very concerned that the carotid bodies are not stimulated by respiratory acidosis as breathing continues to offload carbon dioxide. But, crucially, they fail to appreciate that low arterial $P_{O_{2}}$ provides its intensely dyspnoeic response via the carotid bodies in and of itself. Lest the reader have any doubt regarding the inadequacy of physiological understanding or dubious qualifications of Copeland et al. (2015) as respiratory physiologists, they also state that ‘Altitude hypoxia has similar effects as the hypoxia one gets from breathing inert gases although it is caused by the inability of the lungs to absorb the oxygen in the air rather than a lack of oxygen in the air’. As illustrated below (see last paragraph of this subsection), the effects of altitude hypoxia are most certainly from the low values of $P_{O_{2}}$ in the inspired air and thus lungs, to which the cerebral circulation is consequently exposed.

Copeland et al. (2015) also cited Ernsting (1963) that ‘there was no reported physical discomfort’ and went so far as to opine that ‘low levels (sic) of hypoxia’ produce euphoria and that the anxiety that presents with asphyxiation (a proposed alternative that could be achieved simply by placing a plastic bag over the victim's head) would not be present. The latter point is disingenuous, because Ernsting (1963) did not comment at all on physical comfort or lack thereof. More importantly, those physiologists among us who have studied the effects of breathing nitrogen anoxia know that it is an intensely disturbing and discomforting experience.

With respect to the (forensic) literature dedicated to nitrogen anoxia/asphyxiation in the context of suicide, there are some equally shocking misinformed physiological statements. Belying the observation that climbers without supplemental O_2_ have summitted Mount Everest, where the inspired $P_{O_{2}}$ (∼42 mmHg) is almost exactly equivalent to breathing 6% inspired O_2_ at sea level, Madentzoglou et al. (2013) opine that ‘Death occurs when O_2_ is present in less than 6% of the atmospheric air’. And even more specious is the claim that ‘(Death) can even be delayed and occur at or after 60 minutes if the atmospheric O_2_ stays at 20%’. The fact that millions of humans live at altitudes such as Mexico City, Mexico, Johannesburg, South Africa or high in the Andes and Himalayas, where the inspired $P_{O_{2}}$ is far lower (≤67 mmHg) than provided by 20% of sea‐level atmospheric pressure (i.e., 143 mmHg) further falsifies the statements made by Madentzoglou et al. (2013).

## 5 TECHNIQUES THAT RENDER HUMANS UNCONSCIOUS WITHOUT PAIN

As discussed above, the impact of breathing pure nitrogen on arterial $P_{O_{2}}$, hence brain arterial O_2_ content, is contingent on the lung volume at which the inspirate is switched and on the ensuing ventilation and breathing pattern. These factors introduce delays and variability into the time when consciousness is lost and, by lowering arterial $P_{O_{2}}$, intensely stimulate the carotid bodies, evoking a profound dyspnoea and air hunger. In contrast, sudden occlusion of the cerebral circulation renders the human unconscious in a mere 7–8 s (Nimura et al., 2022; Rossen et al., 1943), without invoking either hyperpnoea or the carotid sinus baroreceptor reflex, as evidenced by lack of major changes in heart rate, blood pressure, myocardial contractility, stroke volume or cardiac output (Mitchell et al., 2012; reviewed by Nimura et al., 2022).

The judo technique of *shime waza* or strangleholds, also used in Brazilian jiu‐jitsu and sometimes referred to as chokes, is designed to elicit a submission within seconds of application. If the judoka opponent does not submit and loses consciousness, upon restoration of carotid artery flow, revival takes ≤12 s. Application of *shime waza* decreases mid‐cerebellar and internal carotid artery blood flow by 80%–90% (Nimura et al., 2022; Reay & Holloway, 1982); brain oxygenation plummets (Haga et al., 2016) and is attended by tonic and clonic convulsions at loss of consciousness, when the EEG demonstrates high‐amplitude slow waves (delta waves) (Ikai et al., 1958; Ogawa et al., 1958; Shibayama & Ebashi, 1978). Loss of consciousness occurs with a reduction of mid‐cerebellar arterial blood flow velocity of >50% (Mitchell et al., 2012; Njemanze, 1992). Despite these anoxic convulsions after fainting, upon restoration of brain blood flow subjects can stand, walk and proceed with their work within 1–2 min after regaining consciousness (Nimura et al., 2022). Repeated fainting consequent to *shime waza* has been considered to be safe and to lack acute or delayed side effects (Matsunaga et al., 2021; Mitchell et al., 2012; Rossen et al., 1943; reviewed by Nimura et al., 2022), although there might be some chronic effects, including adaptive neuroprotection (Stacey et al., 2021). For nearly a century of judo practice and competition, from 1882 to 1979, no deaths attributable to *shime waza* were recorded (Koiwa, 1987), and for jiu‐jitsu exponents, who typically experience more frequent strangulation, no indication of cognitive impairment is evident (Stacey et al., 2021).

As a means to study the impact of acute cerebral anoxia in humans, the Kabat–Rossen–Anderson cuff was developed to increase cervical pressure to 600 mmHg within 0.15 s (Rossen et al., 1943 reviewed by Nimura et al., 2022). By occluding the carotid arteries, inflation of the Kabat–Rossen–Anderson cuff induces acute brain anoxia without affecting breathing or evoking the dyspnoea that attends carotid body stimulation via breathing pure nitrogen. Like *shime waza*, the acute procedure resulting in loss of consciousness is well tolerated, followed by rapid and uneventful recovery upon release, and can be repeated without apparent gross injury to the subjects (Rossen et al., 1943; Smith et al., 2011). This conclusion is also substantiated by the judo (and jiu‐jitsu) communities, in which loss of consciousness from *shime waza* is not uncommon during training and competition and can be followed by a feeling of reperfusion‐induced euphoria. The popularity of judo and especially jiu‐jitsu, despite the regular imposition of strangles/neck chokes (>70% of jiu‐jitsu exponents have been choked more than 100 times, with 28% losing consciousness) attests to the perception that this technique is relatively benign in nature (Stacey et al., 2021; Stellpflug et al., 2020).

Prolonged application of the Kabat–Rossen–Anderson cuff would induce rapid unconsciousness with, for the purposes of execution, the cuff remaining inflated until death was confirmed.

## 6 CONCLUSION

Rather than becoming unconscious within a few breaths and dying within 1 min as stated in the Copeland Report, Smith would have been expected to show signs of severe discomfort and distress with intolerable air hunger for ∼1 min and dying within 5–6 min had he been switched to 100% nitrogen in his mask. Although the exact timing is dependent, in part, upon his breathing pattern and the rate of decreased brain O_2_ supply and metabolism, the eyewitness reports that claim otherwise raise the possibility that the inspired gas was not pure nitrogen, either because the gas cylinder supplying nitrogen did not contain 100% nitrogen or because leaks in the system permitted the entry of O_2_. Like Socrates, we as scientists are obliged continually to challenge the authority of the state when it comes to questions relating to the human body and health, particularly when this authority encompasses deciding over life and death. Regardless of whether one supports the use of state execution as a penalty, this case shows that the reasoning for using nitrogen anoxia as a ‘humane’ method of execution is flawed by physiological and forensic misconceptions and misinformation.

In closing, please be absolutely clear that we consider the death penalty barbaric and unnecessary. We unequivocally oppose its presence in a just society.

## AUTHOR CONTRIBUTIONS

David C. Poole conceived the idea and wrote the first draft of the manuscript with input from Damian M. Bailey, David C. Poole and Damian M. Bailey edited and revised the manuscript. David C. Poole and Damian M. Bailey approved the final version submitted for publication and agree to be accountable for all aspects of the work in ensuring that questions related to the accuracy or integrity of any part of the work are appropriately investigated and resolved. All persons designated as authors qualify for authorship, and all those who qualify for authorship are listed.

## CONFLICT OF INTEREST

D.C.P. is Deputy Editor‐in‐Chief (USA) of Experimental Physiology. D.M.B. is Editor‐in‐Chief of Experimental Physiology and affiliated to the companies FloTBI, Inc. and Bexorg, Inc. focused on the technological development of novel biomarkers of cerebral bioenergetic function and structural damage in humans. D.C.P. and D.M.B. were blinded from the review process and from making any editorial decisions for this manuscript.

## FUNDING INFORMATION

D.C.P. is funded by the Elizabeth Chapin Burke Chair in Health and Human Sciences. D.M.B. is funded by a Royal Society Wolfson Research Fellowship (#WM170007).
